# Comodulation of dopamine and serotonin on prefrontal cortical rhythms: a theoretical study

**DOI:** 10.3389/fnint.2013.00054

**Published:** 2013-08-05

**Authors:** Da-Hui Wang, KongFatt Wong-Lin

**Affiliations:** ^1^Department of Systems Science and National Key Laboratory of Cognitive Neuroscience and Learning, Beijing Normal UniversityBeijing, China; ^2^Intelligent Systems Research Centre, School of Computing and Intelligent Systems, University of UlsterDerry, UK

**Keywords:** dopamine DA, serotonin 5-HT, prefrontal cortical circuit, computational model, selective dopamine and serotonin receptor agonist and antagonist, nonlinear dynamics

## Abstract

The prefrontal cortex (PFC) is implicated to play an important role in cognitive control. Abnormal PFC activities and rhythms have been observed in some neurological and neuropsychiatric disorders, and evidences suggest influences from the neuromodulators dopamine (DA) and serotonin (5-HT). Despite the high level of interest in these brain systems, the combined effects of DA and 5-HT modulation on PFC dynamics remain unknown. In this work, we build a mathematical model that incorporates available experimental findings to systematically study the comodulation of DA and 5-HT on the network behavior, focusing on beta and gamma band oscillations. Single neuronal model shows pyramidal cells with 5-HT1A and 2A receptors can be non-monotonically modulated by 5-HT. Two-population excitatory-inhibitory type network consisting of pyramidal cells with D1 receptors can provide rich repertoires of oscillatory behavior. In particular, 5-HT and DA can modulate the amplitude and frequency of the oscillations, which can emerge or cease, depending on receptor types. Certain receptor combinations are conducive for the robustness of the oscillatory regime, or the existence of multiple discrete oscillatory regimes. In a multi-population heterogeneous model that takes into account possible combination of receptors, we demonstrate that robust network oscillations require high DA concentration. We also show that selective D1 receptor antagonists (agonists) tend to suppress (enhance) network oscillations, increase the frequency from beta toward gamma band, while selective 5-HT1A antagonists (agonists) act in opposite ways. Selective D2 or 5-HT2A receptor antagonists (agonists) can lead to decrease (increase) in oscillation amplitude, but only 5-HT2A antagonists (agonists) can increase (decrease) the frequency. These results are comparable to some pharmacological effects. Our work illustrates the complex mechanisms of DA and 5-HT when operating simultaneously through multiple receptors.

## 1. Introduction

The prefrontal cortex (PFC) plays an essential role in many higher brain functions such as goal-directed behavior, action planning, learning, attention, mnemonic processes, inhibitory control, and task switching (Miller, 2000; Fuster, 2001; Miller and Cohen, 2001; Andrade, 2011b). Neural oscillations in the PFC are suggested to be important for communication within the PFC and with other brain regions, and are suggested to regulate such higher cognitive functions (Wang, 2010; Benchenane et al., 2011).

Neural activities in the PFC are known to be regulated by endogenous neuromodulators. In particular, the neuromodulators dopamine (DA) and serotonin (5-HT) can modulate PFC neuronal excitability, synaptic transmission, plasticity and other electrical and biochemical properties, and hence affect various brain functions and behaviors (de Almeida et al., 2008; Kehagia et al., 2010; Puig and Gulledge, 2011; Rogers, 2011; Puig and Miller, 2012; Tritsch and Sabatini, 2012). DA alone can modulate the PFC in various ways through D1-like (comprising D1 and D5) receptors and D2-like (comprising D2, D3, and D4) receptors expressed on the pyramidal cells and interneurons (Vincent et al., 1993; Gaspar et al., 1995; Vincent et al., 1995; Muly et al., 1998; Neve et al., 2004; Seamans and Yang, 2004; Lapish et al., 2007; de Almeida et al., 2008; Santana et al., 2009). D1-like receptors activation can increase the intrinsic excitability and the input-output gain of PFC pyramidal cell (Henze et al., 2000; Thurley et al., 2008). D1-like receptor is also found to directly depress excitatory interaction between pyramidal cells, increase the excitability of fast-spiking interneurons, and also enhance inhibitory (GABAergic) synaptic transmission (Zhou and Hablitz, 1999; Gao et al., 2001; Gulledge and Jaffe, 2001; Gonzalez-Burgos et al., 2002; Gorelova et al., 2002; Kroner et al., 2007). These can be attributed to D1-like receptors' ability to trigger a variety of ionic channel activities, e.g., enhancement of sodium current, and attenuation of slowly-inactivating potassium currents and glutamate mediated synaptic currents (Yang and Seamans, 1996; Gao et al., 2001; Seamans et al., 2001a; Gonzalez-Islas and Hablitz, 2003; Tseng and O'Donnell, 2004). Activation of D2-like receptors seems to lead to opposite effects of D1-like receptors (Sesack and Bunney, 1989; Yang and Mogenson, 1990; Gulledge and Jaffe, 1998).

The PFC also receives dense 5-HT innervation from the raphe nuclei (Vertes, 1991; Vertes et al., 1999; de Almeida et al., 2008). Although 5-HT can modulate neural activity through seven distinct subtypes of receptors (Hoyer et al., 2002), 5-HT1A and 5-HT2A receptors are abundant in the PFC and seem to be the main contributors. Specifically, about 50–60% of the pyramidal neurons express 5-HT1A and/or 5-HT2A receptors (Pazos and Palacios, 1985; Pompeiano et al., 1992, 1994; Kia et al., 1996; Lopez-Gimenez et al., 1997; Willins et al., 1997; Martin-Ruiz et al., 2001; Santana et al., 2004; de Almeida and Mengod, 2007; Wedzony et al., 2008; Weber and Andrade, 2010), while a subpopulation of pyramidal cells express 5-HT1A or 5-HT2A receptors alone (Amargos-Bosch et al., 2004; Santana et al., 2004; Weber and Andrade, 2010; Andrade, 2011a). Inhibitory interneurons in the PFC also express 5-HT1A or 5-HT2A receptors (Pazos and Palacios, 1985; Willins et al., 1997; Santana et al., 2004; de Almeida and Mengod, 2007; Di Pietro and Seamans, 2007; Puig et al., 2010; Weber and Andrade, 2010). 5-HT1A and 2A receptors seem to act in opposing ways. For example, the activation of 5-HT1A receptors can lead to an increase in potassium conductance, resulting in an inhibitory response of the neuronal membrane potential (Andrade et al., 1986; Beique et al., 2004; Goodfellow et al., 2009), while the activation of 5-HT2A receptors generates an excitatory response by a decrease in the potassium conductance (Zhang and Arsenault, 2005; Andrade, 2011a) or mediating a calcium-sensitive nonspecific cation conductance (Villalobos et al., 2005; Zhang and Arsenault, 2005). *In vivo* and *in vitro* studies demonstrate that 5-HT evokes different response on pyramidal cells: inhibitions, excitations, and biphasic response, but the overall effect is overwhelmingly inhibitory (Puig et al., 2005). In addition to modulating neuronal excitability, 5-HT1A and 5-HT2A receptors can also modulate synaptic transmission. For example, 5-HT1A receptor activation can decrease the function of AMPA (Cai et al., 2002) and NMDA (Cai et al., 2002; Zhong et al., 2008). In contrast, 5-HT2A receptor activation can enhance the function of AMPA (Cai et al., 2002) and NMDA (Yuen et al., 2005). Activation of 5-HT2A receptors inhibits GABA_*A*_ function through phosphorylation of GABA_*A*_ receptors (Feng et al., 2001; Zhong and Yan, 2004).

At the neuronal network level, it has been found that DA injected in the PFC of anesthetized rats enhances hippocampal-prefrontal coherence in the theta band oscillation (Benchenane et al., 2010), which could be due to DA modulating the GABAergic inhibition (Tierney et al., 2008). Blocking D1 receptors has been known to increase alpha and beta band oscillations more in local field potentials for novel than familiar associations (Puig and Miller, 2012). Increasing extracellular DA with genetic polymorphism of dopamine transporter (DAT1) in humans can enhance evoked gamma response to stimulus (Demiralp et al., 2007) 5-HT can also increase the frequency and amplitude of slow waves by promoting the UP states in PFC via activation of 5-HT2A receptors, suggesting an excitatory effect in *in vivo* condition (Puig et al., 2010). 5-HT2A/2C receptor agonist/antagonist has also been found to synchronize/desynchronize frontal cortical oscillations in anesthetized rats (Budzinska, 2009).

Dysregulation of DA and 5-HT in the PFC, and abnormal neural activity levels and oscillations in the PFC are implicated in various mental illnesses such as schizophrenia, attention deficit hyperactivity disorder, depression and addiction (Basar and Guntekin, 2008; Robbins and Arnsten, 2009; Ross and Peselow, 2009; Artigas, 2010; Curatolo et al., 2010; Arnsten, 2011; Meyer, 2012; Noori et al., 2012). Abnormal cortical oscillations can be observed in various neurological and psychiatric disorders, and in particular, disrupted beta (12–30 Hz) and gamma (30–80 Hz) band oscillations are found in schizophrenia, major depression and bipolar disorder (Spencer et al., 2003; Cho et al., 2006; Uhlhaas and Singer, 2006; Basar and Guntekin, 2008; Gonzalez-Burgos and Lewis, 2008; Gonzalez-Burgos et al., 2010; Uhlhaas and Singer, 2010, 2012). For example, schizophrenic patients have enhanced power in the beta2 (16.5–20 Hz) frequency band in the frontal cortex as compared to controls (Merlo et al., 1998; Venables et al., 2009). Beta band oscillation in the frontal cortex in a rat model of Parkinson's disease is also abnormally high compared to controls (Sharott et al., 2005). These mental disorders are usually treated with neuropharmacological drugs that target the DA and/or 5-HT systems (Di Pietro and Seamans, 2007; Bolasco et al., 2010; Poewe et al., 2010; Meltzer and Massey, 2011), which also seem to influence brain rhythms (Kleinlogel et al., 1997; Nichols, 2004; Sharott et al., 2005; Budzinska, 2009).

Although there have been extensive investigations on the modulation of DA and 5-HT on the PFC, little is known about their comodulation effects on the PFC network dynamics and their potential applications in drug treatments (Diaz-Mataix et al., 2005; Di Pietro and Seamans, 2007; Artigas, 2010). In fact, many of the DA and 5-HT induced intracellular signaling pathways overlap (Amargos-Bosch et al., 2004; Santana et al., 2004; Di Pietro and Seamans, 2007; Esposito et al., 2008; Santana et al., 2009), suggesting that DA and 5-HT may cooperatively modulate PFC activity. One notable study has found that coadministration of 5-HT2A antagonist with a D2 antagonist in PFC significantly increase DA release which is greater than that induced by either antagonist alone (Westerink et al., 2001). A recent research has found that co-application of DA and 5-HT can increase the evoked excitability of certain PFC pyramidal cells (the gain of the neuronal input-output response) more than when either was applied alone, while the activities of other pyramidal cells get more suppressed (Di Pietro and Seamans, 2011). Furthermore, the same study also shows that prior DA or 5-HT application can potentiate the subsequent effect of the other.

In this work, we integrate the essential available experimental findings into a biologically motivated computational model to provide insights into the possible PFC dynamics caused by the comodulation of DA and 5-HT. The focus will be on tonic DA and 5-HT modulations, and their effects on higher frequency band oscillations.

## 2. Materials and methods

The computational models in this work will implement DA and 5-HT comodulation at the neuronal and synaptic levels. In the following, we shall discuss about the various neuronal constituents of the PFC modulated by DA and 5-HT.

### 2.1. Subgroups of PFC neurons

The modulation of DA and 5-HT on the neuronal activity depends on the specific receptor subtypes and their combinations since they can evoke different intracellular signaling pathways. Therefore, we divide the pyramidal cells and inhibitory interneurons into subgroups according to their expression of receptors (see Table 1). For simplicity, we omit neurons that do not express DA or 5-HT receptors. We also ignore pyramidal cells expressing DA or 5-HT receptors only, and those co-expressing 5-HT2A and D1-like receptors due to the relatively lower expression of 5-HT2A receptors. Thus, we consider 4 subgroups of pyramidal cells expressing the following combinations of receptors: D1+5-HT1A, D2+5-HT1A, D1+5-HT1A+5-HT2A, and D2+5-HT1A+5-HT2A. For inhibitory interneurons, we also consider 4 subgroups: D1+5-HT1A, D2+5-HT1A, D1+5-HT2A, D2+5-HT2A.

**Table 1:** Percentage of prefrontal cortical neurons expressing DA and 5-HT receptor subtypes^*^.

		**Pyr**			**Int**	
5-HT1A+5-HT2A		38–47%			- - -
5-HT1A		44–59%			20–28%
5-HT2A		3–7%			11–34%	
	II-III	V	VI	II-III	V	VI
D1	19%	21%	38%	28%	30%	38%
D2	5%	25%	13%	5%	8%	17%

* Data adapted from references, Gaspar et al., 1995; Amargos-Bosch et al., 2004; Santana et al., 2004, 2009; Andrade, 2011a; Puig, 2011.

The instantaneous population firing rate for each neuronal subgroup follows the established dynamics (Wilson and Cowan, 1972; Dayan and Abbott, 2001; Murphy and Miller, 2009):

(1) $$\tau_{i}\frac{dr_{i}}{dt}=-r_{i}+f_{i}(I_{i,\text{syn}})$$

where *i* is the index for the subgroup of neurons. For pyramidal cells, *i* = 1 to 4 denote the subpopulation expressing D1+5HT1A, D2+5HT1A, D1+5HT1A+5-HT2A, and D2+5HT1A+5-HT2A, respectively. For interneurons, *i* = 5 to 8 denote the subgroup neurons expressing D1+5HT1A, D1+5HT2A, D2+5HT1A, and D2+5HT2A, respectively. τ_*i*_ is the neuronal membrane time constant, set at 10 ms for pyramidal cells and 15 ms for inhibitory neurons (McCormick et al., 1985). *I*_*i*, syn_ is the total synaptic currents to the *i*-th subgroup. The activation function *f*_*i*_(*I*_*i*, syn_) follows the established form (Eckhoff et al., 2011):

(2) $$f(I_{\text{syn}})=\frac{CI_{\text{syn}}-I_{L}}{1-\text{exp}[-g(CI_{\text{syn}}-I_{L})]+(CI_{\text{syn}}-I_{L})/r_{\max}}$$

where *r*_max_ denotes the saturated firing rate, *r*_max_ is 80 Hz for pyramidal cells, and 120 Hz for inhibitory neurons. *C* is the gain and set as 300 Hz/nA for pyramidal cells and 500 Hz/nA for inhibitory interneurons. *I*_*L*_ is associated with the membrane leakage current and its value is set at 150 Hz for pyramidal cells and 180 Hz for inhibitory interneurons. The curvature of the activation function *g* is 0.2 Hz^−1^.

### 2.2. Modulation of neuronal excitability

In DA modulation, although D1 and D2 receptors can act on different signaling transduction pathways, they can effectively attain opposite effects (e.g., D1 activate protein kinase A while D2 receptors inactivate it) (Trantham-Davidson et al., 2004; Beaulieu and Gainetdinov, 2011; Tritsch and Sabatini, 2012). Therefore, we model the modulation of D1 (D2) on neuronal activity by increasing (decreasing) the gain factor *C* and decreasing (increasing) the leakage factor *I*_*L*_ of the input-output function (Thurley et al., 2008).

With regard to 5-HT modulation, experiments have shown that 5-HT1A can hyperpolarize the neurons through the activation of G protein-gated inwardly rectifying K^+^ channels, while 5-HT2A activation can induce slow membrane depolarization and inhibition of the slow after-hyperpolarization which increases membrane excitability (Andrade et al., 1986; Beique et al., 2004; Goodfellow et al., 2009; Andrade, 2011a). 5-HT1A receptors are often localized on the axon initial segment and soma of pyramidal neurons where they act to suppress action potential generation, while 5-HT2A receptors are abundant in apical dendrites where they can amplify the synaptic current (Amargos-Bosch et al., 2004; Santana et al., 2004). 5-HT2A receptors are also found to increase the gain of the input-output relationship of pyramidal neurons (Zhang and Arsenault, 2005). Therefore, the modulation of 5-HT on neuronal activity can be modeled by an increase in the leakage factor *I*_*L*_ (for 5-HT1A) and increase of the gain factor *C* (for 5-HT2A).

The activation of D1, D2, 5-HT1A and 5-HT2A receptors are concentration dependent (Trantham-Davidson et al., 2004; Hurley, 2006; Solt et al., 2007). For simplicity, we apply the sigmoid function to describe the concentration dependent modulation of DA and 5-HT on the gain and leak factors (Tables 2 and 3), similar to previous work (Fellous and Linster, 1988; Scheler, 2004). In these formulae, [DA]_1_ denotes the half maximal effective concentration (EC_50_) of DA for D1 receptor, and [DA]_2_ is the EC_50_ of DA for D2 receptor. Similarly, [5−HT]_1_ and [5-HT]_2_ are the EC_50_ for 5-HT1A and 5-HT2A receptors, respectively. In brief, we depict the modulation of DA and 5-HT on neuronal activity by changing the activation function through multiplying *C* with a gain factor shown in Table 2, and *I*_*L*_ with the leakage factor shown in Table 3.

**Table 2:** **Gain factor for the neuronal subgroups**.

**Neuron**	**Receptors**	**Gain factor**
Pyr1	D1+5-HT1A	$\left(1+\frac{\varepsilon_{1}}{1+e^{-\beta_{1}([\text{DA}]-{\left[\text{DA}\right]}_{1})}}\right)$
Pyr2	D2+5-HT1A	$\left(1-\frac{\varepsilon_{2}}{1+e^{-\beta_{2}([\text{DA}]-{\left[\text{DA}\right]}_{2})}}\right)$
Pyr3	D1+5-HT1A+5-HT2A	$\left(1+\frac{\varepsilon_{1}}{1+e^{-\beta_{1}([\text{DA}]-{\left[\text{DA}\right]}_{1})}}\right)\times \left(1+\frac{\delta_{2}}{1+e^{-\alpha_{2}([5\text{-HT}]-{\left[5\text{-HT}\right]}_{2})}}\right)$
Pyr4	D2+5-HT1A+5-HT2A	$\left(1-\frac{\varepsilon_{2}}{1+e^{-\beta_{2}([\text{DA}]-{\left[\text{DA}\right]}_{2})}}\right)\times \left(1+\frac{\delta_{2}}{1+e^{-\alpha_{2}{\left([5\text{-HT}]-5\text{-HT}\right]}_{2})}}\right)$
Int1	D1+5-HT1A	$\left(1+\frac{\varepsilon_{1}}{1+e^{-\beta_{1}([\text{DA}]-{\left[\text{DA}\right]}_{1})}}\right)$
Int2	D1+5-HT2A	$\left(1+\frac{\varepsilon_{1}}{1+e^{-\beta_{1}([\text{DA}]-{\left[\text{DA}\right]}_{1})}}\right)\times \left(1+\frac{\delta_{2}}{1+e^{-\alpha_{2}([5\text{-HT}]-{\left[5\text{-HT}\right]}_{2})}}\right)$
Int3	D2+5-HT1A	$\left(1-\frac{\varepsilon_{2}}{1+e^{-\beta_{2}([\text{DA}]-{\left[\text{DA}\right]}_{2})}}\right)$
Int4	D2+5-HT2A	$\left(1-\frac{\varepsilon_{2}}{1+e^{-\beta_{2}([\text{DA}]-{\left[\text{DA}\right]}_{2})}}\right)\times \left(1+\frac{\delta_{2}}{1+e^{-\alpha_{2}([5\text{-HT}]-{\left[5\text{-HT}\right]}_{2})}}\right)$

Pyr, Pyramidal cells; Int, Interneurons.

See text for parameter values.

**Table 3:** **Leakage factor for the neuronal subgroups**.

**Neuron**	**Receptors**	**Leakage factor (*I*_*i*_)**
Pyr1	D1+5-HT1A	$\left(1-\frac{{\bar{\varepsilon }}_{1}}{1+e^{-{\bar{\beta }}_{1}([\text{DA}]-{\left[\text{DA}\right]}_{1})}}\right)\times \left(1+\frac{{\bar{\delta }}_{1}}{1+e^{-{\bar{\alpha }}_{1}([5\text{-HT}]-{\left[5\text{-HT}\right]}_{1})}}\right)$
Pyr2	D2+5-HT1A	$\left(1+\frac{{\bar{\varepsilon }}_{2}}{1+e^{-{\bar{\beta }}_{2}([\text{DA}]-{\left[\text{DA}\right]}_{2})}}\right)\times \left(1+\frac{{\bar{\delta }}_{1}}{1+e^{-{\bar{\alpha }}_{1}([5\text{-HT}]-{\left[5\text{-HT}\right]}_{1})}}\right)$
Pyr3	D1+5-HT1A+5-HT2A	$\left(1-\frac{{\bar{\varepsilon }}_{1}}{1+e^{-{\bar{\beta }}_{1}([\text{DA}]-{\left[\text{DA}\right]}_{1})}}\right)\times \left(1+\frac{{\bar{\delta }}_{1}}{1+e^{-{\bar{\alpha }}_{1}([5\text{-HT}]-{\left[5\text{-HT}\right]}_{1})}}\right)$
Pyr4	D2+5-HT1A+5-HT2A	$\left(1+\frac{{\bar{\varepsilon }}_{2}}{1+e^{-{\bar{\beta }}_{2}([\text{DA}]-{\left[\text{DA}\right]}_{2})}}\right)\times \left(1+\frac{{\bar{\delta }}_{1}}{1+e^{-{\bar{\alpha }}_{1}([5\text{-HT}]-{\left[5\text{-HT}\right]}_{1})}}\right)$
Int1	D1+5-HT1A	$\left(1-\frac{{\bar{\varepsilon }}_{1}}{1+e^{-{\bar{\beta }}_{1}([\text{DA}]-{\left[\text{DA}\right]}_{1})}}\right)\times \left(1+\frac{{\bar{\delta }}_{1}}{1+e^{-{\bar{\alpha }}_{1}([5\text{-HT}]-{\left[5\text{-HT}\right]}_{1})}}\right)$
Int2	D1+5-HT2A	$\left(1-\frac{{\bar{\varepsilon }}_{1}}{1+e^{-{\bar{\beta }}_{1}([\text{DA}]-{\left[\text{DA}\right]}_{1})}}\right)$
Int3	D2+5-HT1A	$\left(1+\frac{{\bar{\varepsilon }}_{2}}{1+e^{-{\bar{\beta }}_{2}([\text{DA}]-{\left[\text{DA}\right]}_{2})}}\right)\times \left(1+\frac{{\bar{\delta }}_{1}}{1+e^{-{\bar{\alpha }}_{1}([5\text{-HT}]-{\left[5\text{-HT}\right]}_{1})}}\right)$
Int4	D2+5-HT2A	$\left(1+\frac{{\bar{\varepsilon }}_{2}}{1+e^{-{\bar{\beta }}_{2}([\text{DA}]-{\left[\text{DA}\right]}_{2})}}\right)$

Pyr, Pyramidal cells; Int, Interneurons. See text for parameter values.

The concentrations of DA and 5-HT in Tables 2 and 3 can be inferred from experiments such as those using microdialysis and voltammetry techniques. It is shown that the basal extracellular DA and 5-HT concentrations in the PFC is about ~0.2–2.5 nM/L in resting condition, and can increase by as much as 10–200% when performing behavioral tasks (Adell et al., 1991; Watanabe et al., 1997; Lena et al., 2005; Winstanley et al., 2006; Rogoz and Golembiowska, 2010; Seeman, 2010; Staiti et al., 2011; van Dijk et al., 2012).

D1- and D2-like receptors can have a high affinity state with a binding constant around the nM/L level or a low affinity state with a binding constant around the μM/L level (Richfield et al., 1989). Since we are studying only tonic concentration levels, we shall only focus on the high affinity receptors, by assuming that low affinity ones are activated more in the phasic or evoked mode, e.g., during behavioral tasks. In particular, we assume that the high affinity D1- and D2-like receptors are sensitive within a range of 0–50 nM/L with different EC_50_ values. We chose [DA]_1_ = 4 nM/L and [DA]_2_ = 8 nM/L (Koshkina, 2006), suggesting lower [DA] activates D1 receptor only while higher [DA] activates both D1 and D2 receptors, similar to the observed activation order of these receptors depending on DA concentration (Trantham-Davidson et al., 2004).

Similarly, 5-HT1A and 5-HT2A receptors can also operate in high-affinity and low-affinity states (Glennon et al., 1998; Watson et al., 2000). In this work, we assume that 5-HT1A and 5-HT2A receptors operate at high-affinity state since tonic 5-HT concentration at the nM/L level is far below the affinity of 5-HT for the low agonist affinity state (Watson et al., 2000), and we vary the 5-HT concentration within the range 0–5 nM/L. The affinity of 5-HT for 5-HT1 is higher than most of other subtype of 5-HT receptors, and lower concentrations favor 5-HT1A receptor activation (Ramage, 2010). One recent research shows that 5-HT1A receptors has lower EC_50_ than that of 5-HT2A receptors in concentration-electrophysiological response relationship (Goodfellow and Lambe, 2009). Thus, we adopt a lower affinity for 5-HT2A receptors than that of 5-HT1A. In particular, we assign [5-HT]_1_ = 1 nM and [5-HT]_2_ = 2 nM. For brevity, we shall drop the 1/L units for the dopamine and serotonin concentrations.

**Figure 1:**
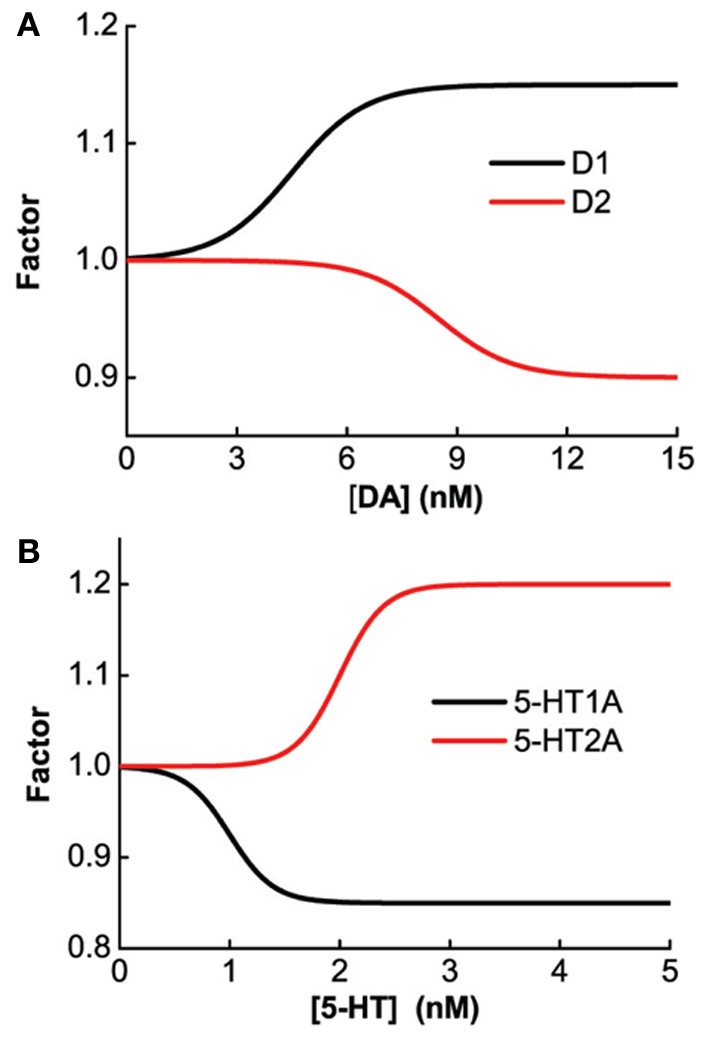
**Concentration dependent modulation factor of DA and 5-HT.** Black (red) line: gain modulation factor due to D1 (D2) receptors as a function of DA concentration **(A)**; modulation factor due to 5-HT1A (5-HT2A) receptors as a function of 5-HT concentration **(B)**.

The specific gain modulation parameter values are as follows: ε_1_ = 0.15 for D1, ε_2_ = 0.1 for D2, and δ_2_ = 0.2 for 5-HT2A. The parameter values reflecting the curvature of gain modulation are chosen as: β_1_ = β_2_ = 1/nM for D1 and D2, and α_2_ = 4/nM for 5-HT2A. The parameters describing the amplitude of leak modulation are chosen as: ${\bar{\varepsilon }}_{1}=0.15$ for D1, ${\bar{\varepsilon }}_{2}=0.1$ for D2, ${\bar{\delta }}_{1}=0.15$ for 5-HT1A. The parameters reflecting the curvature of leak modulation are chosen as: ${\bar{\beta }}_{1}=1/nM$ for D1, ${\bar{\beta }}_{2}=1/nM$ for D2, ${\bar{\delta }}_{1}=4/nM$ for 5-HT1A. The modulation factors due to D1, D2, 5-HT1A and 5-HT2A receptors are shown in Figure 1.

### 2.3. Synaptic currents

The synaptic currents are mediated by AMPA, NMDA, and GABA receptors. We adopt an all-to-all connectivity among neuronal subgroups, thus, the synaptic currents to one neuron in the *i*-th subgroup can be approximated by summing all presynaptic neurons (*N*_*j*_) and normalized by the neurons in the *i*-th subgroup (*N*_*i*_):

(3) $$I_{i,\text{ syn}}=\left(\sum_{j=1}^{4}\left(G_{A,ij}S_{A,j}+G_{N,ij}S_{N,j}\right)r_{j}-\sum_{j=5}^{8}G_{G,ij}S_{G,j}r_{j}\right)\frac{N_{j}}{N_{i}}+\tau_{A}G_{A,\text{ext},\;i}r_{\text{ext}}$$

where the term τ_*A*_*G*_*A*, ext, *i*_*r*_ext_ describes the constant part of the AMPA mediated background Poisson input with rate *r*_ext_ (2.4 KHz) (Wong and Wang, 2006). Unlike (Wong and Wang, 2006; Eckhoff et al., 2011), we do not include synaptic noise into the model as we find that noise does not significantly affect our results (not shown).

*G*_*X*, *ij*_ in Equation (3) denotes the averaged coefficient or strength of the synaptic currents mediated by receptors *X* (*A* for AMPA, *N* for NMDA, and *G* for GABA) from neuron *j* to *i*. Their values are constrained by the experimentally observed neural circuit oscillation frequencies and are assigned as: *G*_*A*, *PP*_ = 4.42 nA, *G*_*A*, *IP*_ = 4.21 nA, *G*_*N*, *PP*_ = 0.10 nA, *G*_*N*, *IP*_ = 0.83 nA, *G*_*G*, *PI*_ = 2.275 nA, *G*_*G*, *II*_ = 1.75 nA, *G*_*A*, ext, *P*_ = 0.0929 nA, and *G*_*A*, ext, *I*_ = 0.0716 nA. *S*_*X*, *j*_ is the averaged synaptic gating variable mediated by receptors *X* expressing on neuron in the *j*-th subgroup and follows the established dynamical forms (Brunel and Wang, 2001; Wong and Wang, 2006; Eckhoff et al., 2011):

(4) $$\frac{dS_{A,j}}{dt}=-\frac{S_{A,j}}{\tau_{A}}+\frac{r_{j}}{1000}$$

(5) $$\frac{dS_{N,j}}{dt}=-\frac{S_{N,j}}{\tau_{N}}+0.641(1-S_{N,\;j})\frac{r_{j}}{1000}$$

(6) $$\frac{dS_{G,j}}{dt}=-\frac{S_{G,j}}{\tau_{G}}+\frac{r_{j}}{1000}$$

where τ_*A*_ = 2 ms, τ_*N*_ = 100 ms, and τ_*G*_ = 10 ms are the decay time constants for AMPA, NMDA, and GABA receptors, respectively. The fraction *N*_*j*_/*N*_*i*_ can be approximated according to the experimental observations on the DA and 5-HT receptors distribution (Pazos and Palacios, 1985; Amargos-Bosch et al., 2004; Beique et al., 2004; Santana et al., 2004; de Almeida and Mengod, 2007; Santana et al., 2009). Based on experimental observations (see Table 1), we assume that the ratio for pyramidal cells expressing D1 to D2 receptors is approximately 25:15%, and the ratio for interneurons expressing D1 to D2 receptors is approximately 30:10%. We also assume that the ratio for pyramidal cells or interneurons solely expressing 5-HT1A receptors to that those expressing 5-HT1A and 5-HT2A receptors is approximately 50:50%. Moreover, pyramidal cells expressing D2 receptors are often found in apposition with GABAergic cells not expressing D2 receptors, leading to the synaptic currents from inhibitory neurons expressing D2 receptors smaller than those from inhibitory neurons expressing D1 receptors (de Almeida and Mengod, 2007). So we specify the fraction from D2 expressing interneurons to D2 expressing pyramidal cells as a fifth of that from D1 expressing interneurons. Table 4 lists the above-mentioned fractions, where the fraction from the *j*-th to *i*-th subgroup is the value at the *i*-th row and *j*-th column. When simulating the two-population model, we ignore other subgroups by setting the irrelevant connections to zero.

**Table 4:** **Fraction of neuronal subgroups**.

	**Pyr1**	**Pyr2**	**Pyr3**	**Pyr4**	**Int1**	**Int2**	**Int3**	**Int4**
Pyr1	1	$\frac{3}{5}$	1	$\frac{3}{5}$	$\frac{1}{2}$	$\frac{1}{2}$	$\frac{3}{10}$	$\frac{3}{10}$
Pyr2	$\frac{5}{3}$	1	$\frac{5}{3}$	1	$\frac{5}{6}$	$\frac{5}{6}$	$\frac{1}{6}$	$\frac{1}{6}$
Pyr3	1	$\frac{3}{5}$	1	$\frac{3}{5}$	$\frac{1}{2}$	$\frac{1}{2}$	$\frac{3}{10}$	$\frac{3}{10}$
Pyr4	$\frac{5}{3}$	1	$\frac{5}{3}$	1	$\frac{5}{6}$	$\frac{5}{6}$	$\frac{1}{6}$	$\frac{1}{6}$
Int1	2	$\frac{6}{5}$	2	$\frac{6}{5}$	1	1	$\frac{3}{5}$	$\frac{3}{5}$
Int2	2	$\frac{6}{5}$	2	$\frac{6}{5}$	1	1	$\frac{3}{5}$	$\frac{3}{5}$
Int3	$\frac{10}{3}$	2	$\frac{10}{3}$	2	$\frac{5}{3}$	$\frac{5}{3}$	1	1
Int4	$\frac{10}{3}$	2	$\frac{10}{3}$	2	$\frac{5}{3}$	$\frac{5}{3}$	1	1

Pyr, Pyramidal cells; Int, Interneurons.

### 2.4. Modulation of synaptic transmission

The synaptic current coefficients or strengths, *G*_*X*, *ji*_, can be modulated by DA and 5-HT through the activation of D1, D2, 5-HT1A, and 5-HT2A receptors. Studies have demonstrated that D1-like receptors can enhance AMPA, NMDA, and GABA mediated synaptic currents or receptor expression, while D2-like receptors decrease them (Seamans et al., 2001b; Gorelova et al., 2002; Thurley et al., 2008). Similar to the modulation of DA on synaptic currents, 5-HT also bidirectionally modulates the synaptic currents through the activation of different receptors: 5-HT1A receptors reduce AMPA and NMDA mediated currents (Cai et al., 2002; Zhong et al., 2008), while 5-HT2A receptors increase them. Activation of 5-HT2A receptors on pyramidal cells can reduce GABA mediated currents (Feng et al., 2001) while 5-HT1A receptors may suppress the presynaptic GABAergic release in interneurons (Yan, 2002).

We adopt a scalar, sigmoid function factor for the modulation of synaptic current coefficients (Fellous and Linster, 1988; Scheler, 2004). In principle, as all 4 considered receptors can modulate the three (AMPA, NMDA and GABA mediated) synaptic currents, we have 12 possible modulation factors (Table 5). The parameters λ_*ij*_ and κ_*ij*_ depict the modulatory effects of the *i*-th receptor on the *j*-th synaptic type. We set the amplitude λ_*ij*_ = 0.2 and the curvature κ_*ij*_ = 1/nM for *i* = 1, 2 and κ_*ij*_ = 4/nM for *i* = 3, 4.

**Table 5:** **Modulation factors of DA and 5-HT on synaptic currents**.

**AMPA**	**NMDA**	**GABA**
$1+\frac{\lambda_{11}}{1+e^{-\kappa_{11}([\text{DA}]-{\left[\text{DA}\right]}_{1})}}$	$1+\frac{\lambda_{12}}{1+e^{-\kappa_{12}([\text{DA}]\;-{\left[\text{DA}\right]}_{1})}}$	$1+\frac{\lambda_{13}}{1+e^{-\kappa_{13}([\text{DA}]\;-{\left[\text{DA}\right]}_{1})}}$
$1-\frac{\lambda_{21}}{1+e^{-\kappa_{21}([\text{DA}]\;-{\left[\text{DA}\right]}_{2})}}$	$1-\frac{\lambda_{22}}{1+e^{-\kappa_{22}([\text{DA}]\;-{\left[\text{DA}\right]}_{2})}}$	$1-\frac{\lambda_{23}}{1+e^{-\kappa_{23}([\text{DA}]\;-{\left[\text{DA}\right]}_{2})}}$
$1-\frac{\lambda_{31}}{1+e^{-\kappa_{31}([5\text{-HT}]-{\left[5\text{-HT}\right]}_{1})}}$	$1-\frac{\lambda_{32}}{1+e^{-\kappa_{32}([5\text{-HT}]-{\left[5\text{-HT}\right]}_{1})}}$	$1-\frac{\lambda_{33}}{1+e^{-\kappa_{33}([5\text{-HT}]-{\left[5\text{-HT}\right]}_{1})}}$^*^
$1+\frac{\lambda_{41}}{1+e^{-\kappa_{41}([5\text{-HT}]-{\left[5\text{-HT}\right]}_{2})}}$	$1-\frac{\lambda_{42}}{1+e^{-\kappa_{42}([5\text{-HT}]-{\left[5\text{-HT}\right]}_{2})}}$	$1-\frac{\lambda_{43}}{1+e^{-\kappa_{43}([5\text{-HT}]-{\left[5\text{-HT}\right]}_{2})}}$

Top to bottom rows: modulation factors due to D1, D2, 5-HT1A, and 5-HT2A receptors, respectively.

* As activation of 5-HT1A receptors on an interneuron can reduce GABA release, an assumption is made in which GABA-mediated currents are modulated only by presynaptic 5-HT1A receptors on an interneuron and not postsynaptic 5-HT1A receptors.

**Figure 2:**
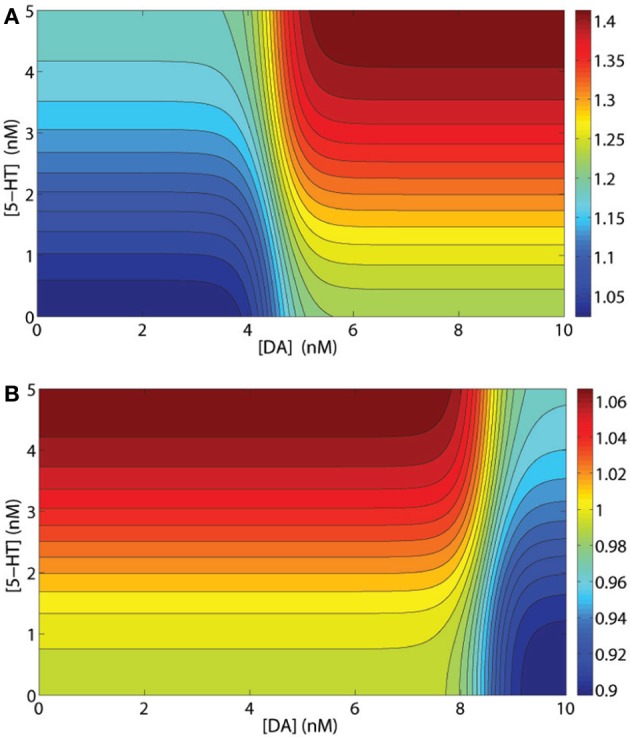
**Examples of synaptic current modulations. (A)** Modulation factor of GABA mediated current from an inhibitory interneuron to a pyramidal cell, both expressing D1 and 5-HT2A receptors. **(B)** Modulation factor of an NMDA- or AMPA-mediated synaptic current by a presynaptic inhibitory neuron expressing D1 and 5-HT1A receptors and a postsynaptic pyramidal cell expressing D1 and 5-HT2A receptors.

Usually, the synaptic modulation factors are determined by the specific types of receptors expressing on the postsynaptic neuron. For example, GABA mediated currents from D1+5-HT2A expressing (presynaptic) interneuron to D1+5-HT2A expressing (postsynaptic) pyramidal cells should be modulated by the factor $\left(1+\frac{\lambda_{23}}{1+e^{-\kappa_{23}([\text{DA}]-{\left[\text{DA}\right]}_{1})}})\times (1-\frac{\lambda_{43}}{1+e^{-\kappa_{43}([5\text{-HT}]-{\left[5\text{-HT}\right]}_{2})}}\right)$. Figure 2A shows that it is more effective to have [DA] and [5-HT] to increase/decrease in the same direction. One exception is the modulation of 5-HT1A on GABA-mediated synaptic currents. As mentioned above, 5-HT1A receptors can modulate the GABA-mediated currents via a presynaptic mechanism by reducing the GABAergic release (Yan, 2002), which effectively reduces the GABA-mediated currents. Thus, we assume that only presynaptic expressing 5-HT1A receptors can modulate GABA-mediated currents by the factor $1-\frac{\lambda_{33}}{1+\text{exp}[-\kappa_{33}([5\text{-HT}]-{\left[5\text{-HT}\right]}_{1})]}$. For example, if the postsynaptic pyramidal neuron expresses D2, 5-HT1A and 5-HT2A receptors, while the presynaptic inhibitory neuron expresses D1 and 5-HT1A receptors, then only the postsynaptic D2 and 5-HT2A receptors and the presynaptic 5-HT1A receptors can modulate this specific synaptic current. The combined modulation factor on the synaptic current is then $\left(1-\frac{\lambda_{33}}{1+\text{exp}[-\kappa_{33}([5\text{-HT}]-{\left[5\text{-HT}\right]}_{1})]})\times (1+\frac{\lambda_{13}}{1-\text{exp}[-\kappa_{13}([\text{DA}]-{\left[\text{DA}\right]}_{2})]})(1+\frac{\lambda_{43}}{1+\text{exp}[-\kappa_{43}([5\text{-HT}]-{\left[5\text{-HT}\right]}_{2})]}\right)$. Figure 2B shows that having DA and 5-HT to increase/decrease in opposite directions provides a more effective modulation effect on this particular synapse.

## 3. Results

In the following, we shall first investigate the effects of DA and 5-HT modulation on individual PFC neurons, and then followed by various coupled excitatory-inhibitory PFC circuits. After that, we investigate how a more realistic heterogeneous multi-population network model is modulated by DA and 5-HT concentration levels and selective receptor agonists/antagonists.

### 3.1. Non-monotonic modulation of 5-HT on pyramidal cell coexpressing 5-HT1A and 5-HT2A receptors

**Figure 3:**
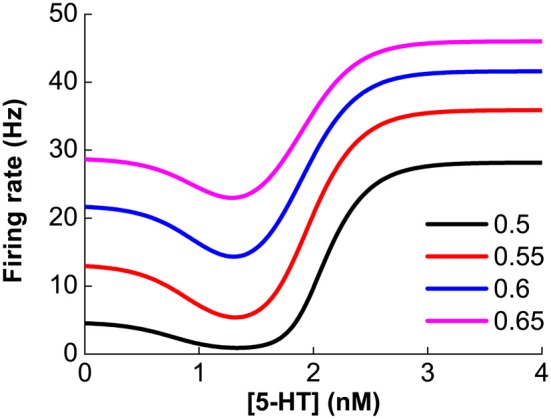
**Non-monotonic modulation of serotonin on the firing rate of a 5-HT1A and 5-HT2A coexpressing pyramidal cell given a fixed synaptic input, *I*_syn_.** Colors denote different constant *I*_syn_ values: 0.5, 0.55, 0.6 and 0.65 nA.

As previously mentioned, our model assumes that D1 and D2 receptors are expressed on distinct neuronal populations. Thus, it is expected that the modulation of DA on single neuronal activity should monotonically depend on the extracellular DA concentration. Similarly, for neurons solely expressing 5-HT1A or 5-HT2A receptors, the neuronal activity will also monotonically depend on extracellular 5-HT concentration. However, for pyramidal cells coexpressing 5-HT1A and 5-HT2A receptors, the modulation of 5-HT is not monotonic. The steady firing rate for such a pyramidal cell is $r=\frac{I}{1-e^{-gI}+I/r_{\max}}$ with $I=(1+\frac{\delta_{2}}{1+e^{-\alpha_{2}([5\text{-HT}]-{\left[5\text{-HT}\right]}_{2})}})\times (1+\frac{\lambda_{41}}{1+e^{-\kappa_{41}([5\text{-HT}]-{\left[5\text{-HT}\right]}_{2})}})C_{P}I_{\text{syn}}-(1+\frac{{\bar{\delta }}_{1}}{1+e^{-{\bar{\alpha }}_{1}([5\text{-HT}]-{\left[5\text{-HT}\right]}_{1})}})I_{L}$. For any constant *I*_syn_, the combined modulation effect is found to be non-monotonic (Figure 3). Lower 5-HT concentration initially decreases the neuronal firing rate because of the activation of the high affinity of 5-HT1A receptors. But higher concentration of 5-HT subsequently increases the neuronal firing rate due to activating the lower affinity 5-HT2A receptors.

### 3.2. Modulation on two-population excitatory-inhibitory neuronal networks

The simplest “canonical” cortical column and its oscillatory behavior can be modeled by an excitatory neuronal population mutually coupled to an inhibitory neuronal population (Wilson and Cowan, 1972). In our model, for every pair of coupled excitatory and inhibitory neuronal populations considered, we set the parameters (i.e., the fractions in Table 4) describing the other (six) populations to be zero. Based on the expression of the various receptors (Tables 2 or 3), there are 4 × 4 = 16 combinations of excitatory and inhibitory neurons in the two-population network model. In general, we find that if the network consists of pyramidal cells which express D2 receptors (Pyr2 or Pyr4), the network cannot attain oscillatory behavior over the ranges of [DA] and [5-HT] explored. However, if the network includes D1-expressing pyramidal cells (Pyr1 or Pyr3), a rich repertoire of dynamical behavior can be produced with varying [DA] and [5-HT], as described below.

**Figure 4:**
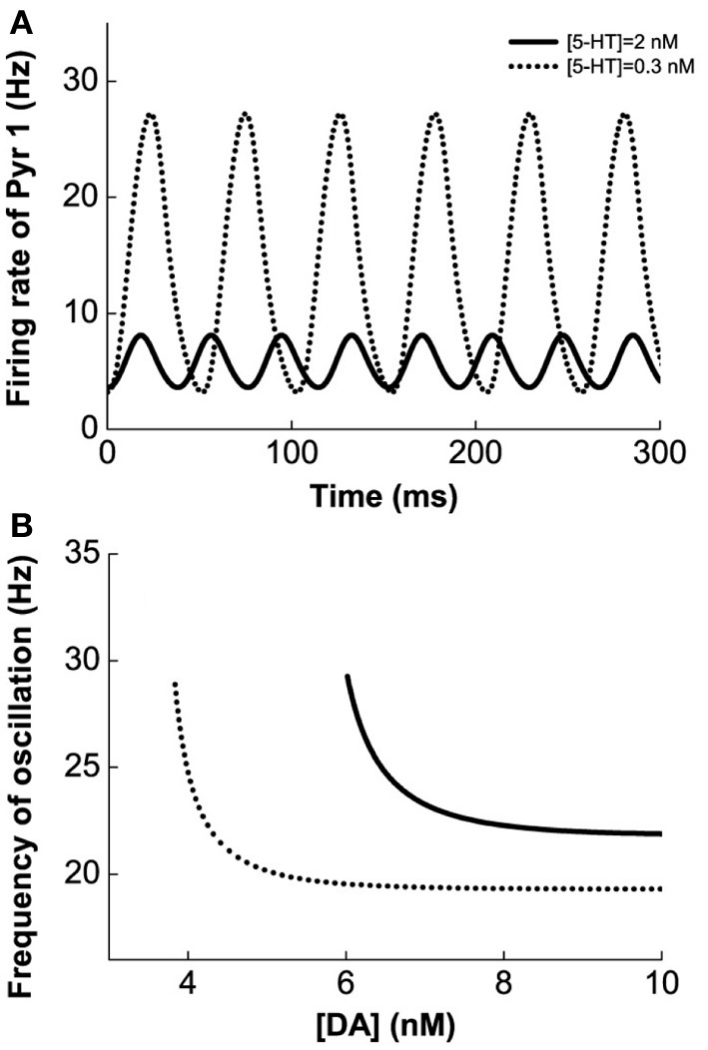
**Modulation of DA and 5-HT on a network consisting of Pyr1-type (D1+5-HT1A) and Int1 (D1+5-HT1A) neurons. (A)** Firing rate time course of pyramidal cells with [DA] = 7 nM and [5-HT] = 0.3 nM (dotted) or 2 nM (solid). **(B)** Oscillation frequency decreases with increasing [DA]. [5-HT] = 0.3 nM (dotted) and 2 nM (solid).

Figure 4 shows the results for the Pyr1-type (D1+5-HT1A) pyramidal cells coupled to Int1-type (D1+5HT1A) interneurons. Figure 4A shows an example of the firing rate time course of Pyr1 neurons for [DA] = 7 nM. Higher [5-HT] level results in lower oscillation amplitude but faster frequency. The firing rate time courses for the inhibitory Int1 populations look similar (not shown). At intermediate [DA], the oscillation frequency decreases with increasing [DA] while remaining within the high beta range (Figure 4B).

**Figure 5:**
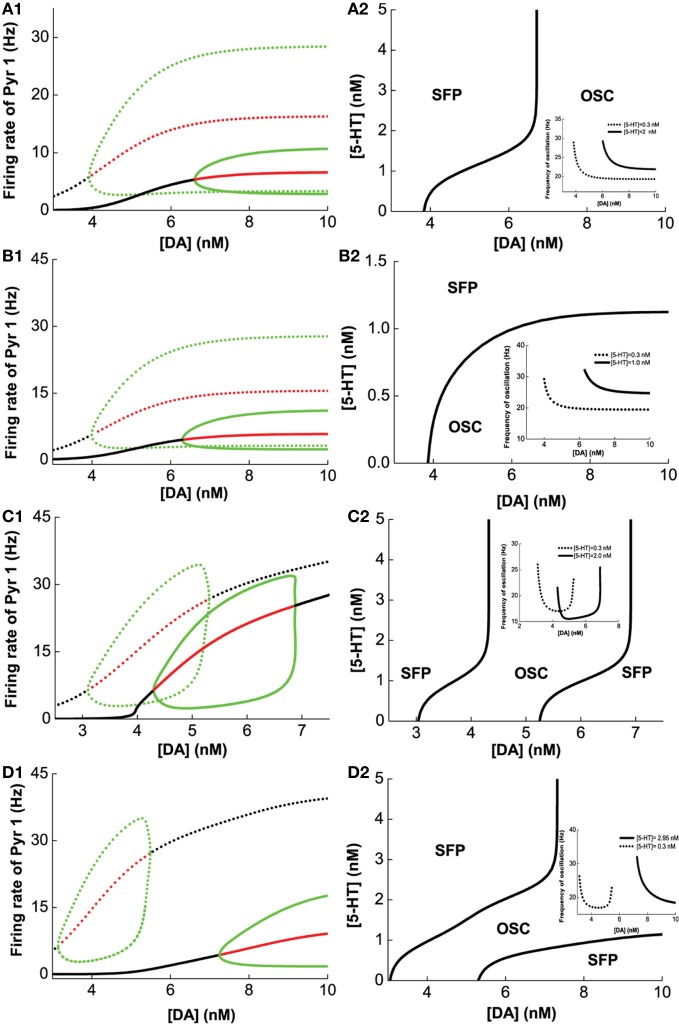
**Modulation of [DA] and [5-**HT**] on excitatory-inhibitory neuronal networks.** Pyr1 (D1+5-HT1A) neurons paired with: **(A)** Int1 (D1+5-1HT1A), **(B)** Int2 (D1+5-HT2A), **(C)** Int3 (D2+5-HT1A), **(D)** Int4 (D2+5-HT2A) neurons. Left: Stability or bifurcation diagrams with respect to [DA] given [5-HT] = 0.3 nM (dotted) or 2, 1, 2,and 2.95 nM 1(solid) for **(A–D)**, respectively. Black (red) lines: stable (unstable) steady states; top/bottom green: maximum/minimum firing rates during oscillation. Right: Phase diagrams with respect to [DA] and [5-HT]. OSC: oscillatory behavior; SFP: only one stable asynchronous steady state (or fixed point). Inset: oscillation frequency vs [DA] with fixed [5-HT] values (in left).

Figure 5A1 (green lines) summarizes the oscillation amplitudes over a range of [DA] levels. The top green lines denote the maximum (top) and minimum (bottom) firing rates during oscillation. The red lines represent collections of unstable steady states (or specifically, unstable fixed points), while the black lines represent that for the asynchronous stable steady states (or stable fixed points). We can also observe that with sufficiently low [DA], oscillations can disappear through a phase transition or bifurcation (specifically, a Hopf bifurcation) (Strogatz, 2001), such that the neurons in the network are tonically and asynchronously firing at stable rates. Clearly, we can see that higher [5-HT] level can laterally shifts the onset of oscillation (bifurcation point) rightward (compare dotted and bold), which means a higher [DA] level is required to maintain the oscillations. Moreover, the range of oscillation amplitudes are also significantly more constrained. Globally, we can also map out the network behavior with respect to the [DA] and [5-HT], i.e., a phase diagram. The phase diagram in Figure 5A2 clearly shows that oscillation behavior can occur when [DA] is sufficiently high (~7 nM) regardless of the [5-HT] level. The inset in Figure 5A2 is a replicate of Figure 4B.

Figures 5B–D show the modulation of [DA] and [5-HT] on the network composing Pry1-type pyramidal cells and rest of the interneuronal types: Int2-type (D1+5-HT2A) inhibitory neurons (B), Int3-type (D2+5-HT1A) inhibitory neurons (C), and Int4-type (D2+5-HT2A) inhibitory neurons (D). For the network consisting of Pyr1 and Int2 neurons, Figure 5B1 shows two examples of the bifurcation diagram for pyramidal cells' firing rate with respect to [DA] given fixed [5−HT] = 0.3 nM (dotted) and [5−HT] = 2.0 nM (solid). The phase diagram (Figure 5B2) shows that the neurons are tonically firing asynchronously given higher [5-HT] and lower [DA], and synchronously firing given smaller [5-HT] and moderately higher [DA]. The oscillation emerges along with increasing [DA] through a Hopf bifurcation. The oscillation amplitude increases and approaches saturation (Figure 5B1), while the oscillation frequency decreases from 30 Hz to approximately 20 Hz with increasing [DA] (inset of Figure 5B2). For the network with D2+5-HT1A inhibitory neurons, only a finite range of [DA] supports oscillation behavior (Figure 5C2). The oscillation emerges and then disappears through Hopf bifurcations with increasing [DA], regardless of the [5-HT] levels. There is also an increase follows by a decrease in oscillation amplitude as [DA] is increased for a given fixed [5-HT] = 0.3 nM (dotted) and [5-HT] = 2.0 nM (solid) (Figure 5C1). Thus a non-monotonic dependence of oscillation frequency on [DA] (Figure 5C2 inset). For the network with Pyr1- and Int4-type (D2 and 5-HT2A) inhibitory neurons, oscillation behavior can be obtained only in a finite range of [DA] if [5-HT] is low ([5-HT] = 0.3 nM) (Figures 5D1,D2), similar to Figure 5C. A similar minimal (maximal) oscillation frequency (amplitude) can be observed (Figure 5D2 inset, dotted). However, for high [5-HT] ([5-HT] > 2 nM) and [DA] (>7 nM), oscillation behavior becomes more easily attainable (bold).

**Figure 6:**
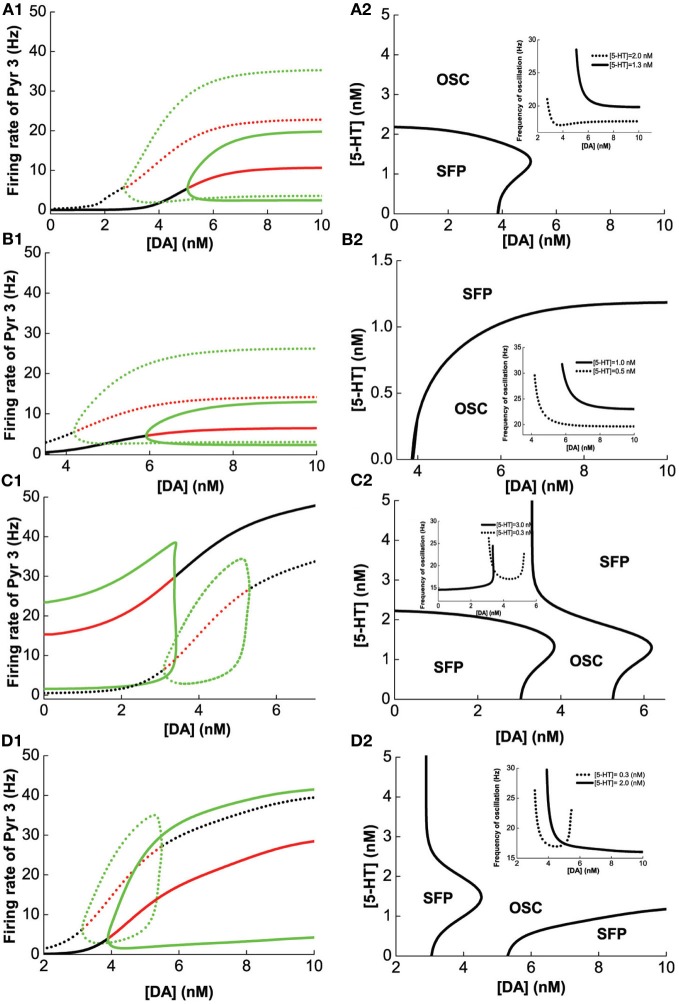
**Modulation of [DA] and [5-**HT**] on excitatory-inhibitory neuronal networks.** Pyr3 (D1+5-HT1A+5-HT2A) neurons paired with: **(A)** Int1 (D1+5-1HT1A), **(B)** Int2 (D1+5-HT2A), **(C)** Int3 (D2+5-HT1A), **(D)** Int4 (D2+5-HT2A) neurons. Left: Stability or bifurcation diagrams with respect to [DA] given [5-HT] = 0.3 nM (dotted) or 2 nM (solid). Right: phase diagrams. Label as in Figure 5.

A similar analysis is done on Pyr3-type(D1+5-HT1A+5-HT2A) pyramidal cells, instead of Pyr1-type. Figure 6 summarizes the analysis of 2-population network of Pyr3 paired individually with the same four (Int1–4) types of inhibitory neurons. The phase diagrams (Figure 6, right) indicate that oscillations cannot be attained if [DA] and [5-HT] levels are sufficiently low. Figures 6A1,A2 (Pyr3 and Int1) look qualitatively similar to Figures 5A1,A2 (Pyr 1 and Int1) except that now there is a finite regime of [5-HT] that does not allow oscillation to occur. Figures 6B1,B2 (Pyr3 and Int2) look qualitatively similar to that of Figures 5B1,B2 (Pyr1 and Int2) throughout the range of [DA] and [5-HT] explored. Figures 6C1,C2 (Pyr3 and Int3) seem to be a hybrid of Figure 6A (Pyr3 and Int1) and Figure 5C (Pyr1 and Int3), with one of the asynchronous regions (Figure 6C2). This implies that when [5-HT] is sufficiently high, oscillation can occur even with very low level of [DA] (<3.5 nM). The network with Pyr3 and Int4 (Figure 6D) looks similar to that of Pyr1 and Int4 (Figure 5D), but the oscillation regime can now occur over a much larger range of [DA] and [5-HT].

We have seen how [DA] and [5-HT] can modulate the network consisting of different combinations of pyramidal cells and inhibitory neurons in Figures 4–6. Taken together, we can make several observations. Firstly, we can observe that pyramidal cells with (excitatory) 5-HT2A receptors can oscillate even with low [DA] (Figures 6A,C,D) as compared to pyramidal cells with no 5-HT2A receptors (Figures 5A,C,D). Secondly, inhibitory neurons with 5-HT2A receptors can enhance inhibition in the circuit, which can cause oscillation to cease (compare Figure 6D2 with Figure 5D2). Thirdly, higher [DA] will inhibit interneurons expressing D2 receptors due to the latter's inhibitory nature upon activation, and as a result, network oscillation will cease (Figures 5C2, D2, 6C2, D2).

### 3.3. Heterogeneous network model

After investigating the DA and 5-HT modulation on various possible two-population excitatory-inhibitory networks, we shall now study the neuromodulation and drug effects on the dynamics of a 8-population network fully connected with neurons expressing all the considered receptor types and their combinations.

#### 3.3.1. 5-HT and DA modulation

**Figure 7:**
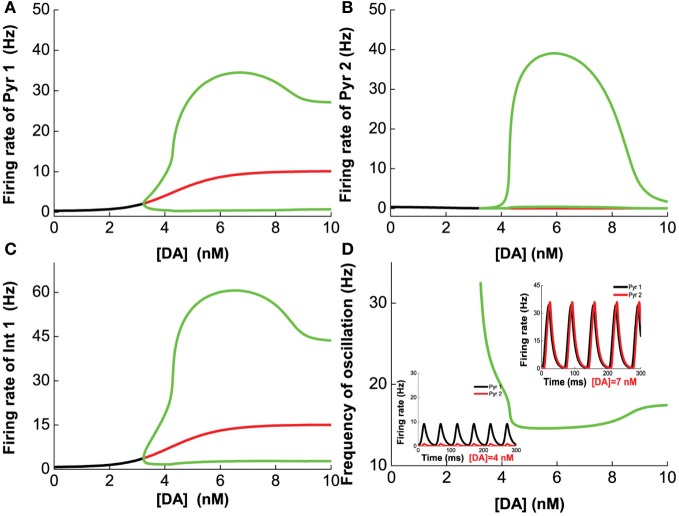
**Dependence of the heterogeneous network behavior on [DA].** Oscillation of the network emerges from a Hopf bifurcation at [DA] = 3.21 nM. **(A–C)** The amplitude of the oscillation increases with increasing [DA] before the activation of D2 receptors reduce **(A,C)** or suppress it **(B)**. **(D)** The frequency of the oscillation decreases with increasing [DA] due to the D1 receptors before it increases slightly again upon activation of D2 receptors. [DA]_1_ = 4 nM, [DA]_2_ = 8 nM, [5-HT]_1_ = 1 nM, [5-HT]_2_ = 2 nM, and [5-HT] = 0.3 nM.

We first vary [DA] to investigate the modulation of DA, fixing [DA]_1_ = 4 nM, [DA]_2_ = 8 nM, [5-HT]_1_ = 1 nM, [5-HT]_2_ = 2 nM, and [5-HT] = 0.3 nM. Oscillation emerges at [DA] = 3.21 nM through a Hopf bifurcation. Due to the higher affinity of the excitatory D1-like receptors than that of inhibitory D2-like receptors, the amplitudes of the neuronal firing rates first increase before reducing or saturating as [DA] increases (Figures 7A–C). This is especially pronounced for pyramidal cells which express D2 receptors (Pyr2, Pyr4, Int3, and Int4), exhibiting an inverted U-shaped modulation (Pyr2 in Figure 7B; Pyr4, Int3 and Int4 not shown due to the modulation on these neuron by DA is similar to that of Pyr2). The oscillation frequency decreases from low gamma to beta band with increasing [DA], before it slightly increases again with further increase in [DA] (Figure 7D). The value of [DA] producing the minimal oscillation frequency (~6 nM) coincides with that of the maximal neuronal firing rates.

**Figure 8:**
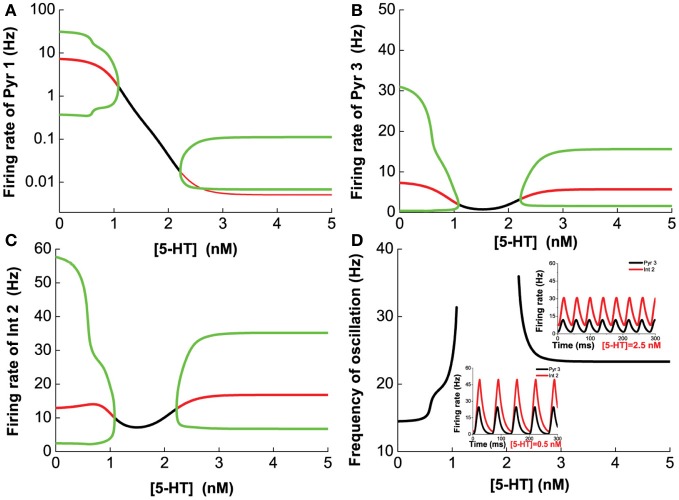
**Dependence of the heterogeneous network behavior on [5-HT].** The network oscillates only for low [5-HT] (<1.08 nM) or high [5-HT] (>2.22 nM). **(A)** Activity of pyramidal cells expressing 5-HT1A is almost totally suppressed by the activation of 5-HT1A when [5-HT] exceeds [5-HT]_1_ (note: log scale). **(B)** Activity of pyramidal cells expressing D1, 5-HT1A, and 5-HT2A receptors. **(C)** Activity of interneurons expressing D1 and 5-HT2A receptors. **(D)** Dependence of the oscillation frequency on [5-HT]. Insets: firing rates of Pyr 3 and Int 2 given [5-HT] = 0.5 nM and 2.5 nM. [DA]_1_ = 4 nM, [DA]_2_ = 8 nM, [5-HT]_1_ = 1 nM, [5-HT]_2_ = 2 nM, and [DA]= 5 nM.

Next, we vary [5-HT] while keeping [DA] fixed at 5 nM, and having [DA]_1_ = 4 nM, [DA]_2_ = 8 nM, [5-HT]_1_ = 1 nM, [5-HT]_2_ = 2 nM, and [DA] = 5 nM. Figure 8 shows that 5-HT modulates the network activity in an interesting manner. The network oscillates either at a low or high [5-HT] level, while intermediate [5-HT] level (within the 1.08–2.22 nM range) leads to asynchronous tonic stable activity (Figures 8A–C). The underlying reason for such a phenomenon is due to the different affinities of 5-HT1A and 5-HT2A receptors. This intermediate tonic stable state may not arise if [5-HT]_1_ > [5-HT]_2_. In fact, we have observed such multiple oscillation regimes in the simpler two-population excitatory-inhibitory network model (Figures 5B2,C2, 6C2,D2). For pyramidal cells without 5-HT2A receptors (Pyr1 and Pyr2), its activity is almost fully suppressed by 5-HT1A receptor inhibition (Figure 8A; Pyr2 not shown). The slight increase in activity with oscillation is indirectly activated by other neuronal subgroups (e.g., excitation from oscillating Pyr3-type neurons; Figure 8B). The frequency of the oscillation increases with increasing [5-HT] before the latter reaches a Hopf bifurcation point (1.08 nM), after which the oscillation ceases. When [5-HT] exceeds a larger critical value (2.22 nM), the frequency decreases toward a stable value of about 24 Hz (Figure 8D). It should be noted that the activations of neurons at high levels of [5-HT] are observed only for Pyr3-type (D1+5-HT1A+5-HT2A) pyramidal cells and Int2-type (D1+5-HT2A) inhibitory neurons, while the other types of neurons are inhibited (not shown).

**Figure 9:**
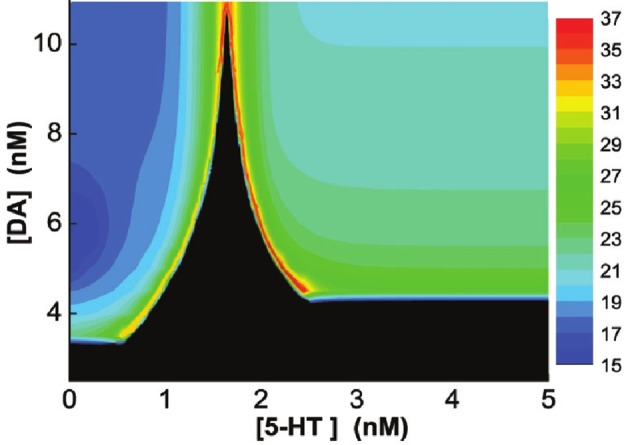
**Heterogeneous network behavior with respect to [DA] and [5-HT].** Black region: Only asynchronous tonic stable firing states. Above the regions, oscillations can occur. Color bar denotes the oscillation frequencies (from 15 to 36 Hz) which depend on the combination of [5-HT] and [DA] levels. For [DA] > 10.946 nM, there exists an optimal [5-HT] value where the network can attain the maximum frequency oscillation.

Finally, we vary [DA] and [5-HT] simultaneously, and find that if [DA] is above a certain value (10.946 nM), the network always oscillates for any [5-HT] level (Figure 9A). Specifically, there exist a Λ-shaped green curve in Figure 9 below which the network cannot support oscillatory activity (black region), while above it oscillation occurs. Although the oscillation frequencies generally decreases with increasing [DA] levels, they stay around the same range (Figure 9). Moreover, the frequency of the oscillation non-monotonically depends on [5-HT]; increasing before [5-HT] exceeds the left branch of the Λ shape in the phase diagram, and then decreasing after [5-HT] exceeds the right branch of the Λ shape (Figure 9). When [DA] > 10.946 nM, there exists an optimal [5-HT] value where the network can attain the maximum frequency oscillation. The peak of the frequency lies in the gamma range, which is often observed during attentional processing (Benchenane et al., 2011). Interestingly, when [DA] is smaller than 4.1 nM, there is only a narrow finite range of [5-HT] values that supports oscillatory behavior.

#### 3.3.2. 5-HT and DA receptor selective agonist/antagonist

Having observed how the PFC network model can be modulated by [DA] and [5-HT], we shall now investigate the influence of DA and 5-HT receptors selective agonist or antagonist. A selective agonist (antagonist) is a drug that can activate (block) a specific receptor without affecting other receptors. Our model can mimic the effect of receptor selective agonist or antagonist by decreasing or increasing the half maximal effective concentration of the receptors, namely, [DA]_1_ for D1, [DA]_2_ for D2, [5-HT]_1_ for 5-HT1A, or [5-HT]_2_ for 5-HT2A receptors (Lambert, 2004; Golan et al., 2007).

**Figure 10:**
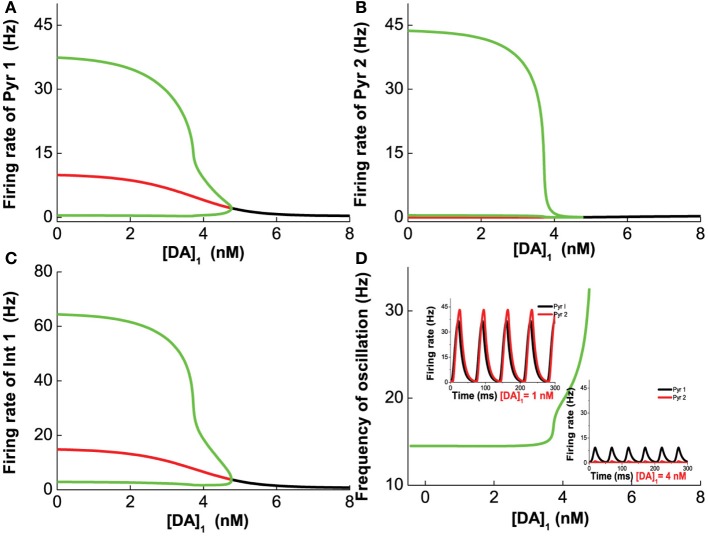
**D1 selective agonist/antagonist on the heterogeneous network.** Increasing [DA]_1_ will decrease the amplitude of oscillation but increase the frequency of oscillation. **(A)** Dependence of firing rate of pyramidal cell (D1+5-HT1A) on [DA]_1_. The amplitude of the oscillation, the difference between the green lines, decreases with increasing [DA]_1_. **(B)** Dependence of firing rate of pyramidal cell (D2+5-HT1A) on [DA]_1_. **(C)** The firing rate of interneurons (D1+5-HT1A) depends on [DA]_1_. **(D)** Frequency of oscillation increases with the increase of [*DA*]_1_ before the agonist/antagonist shuts off the oscillation. Insets: firing rates of two types of pyramidal cells over time with [DA]_1_ = 1 nM (left), and [DA]_1_=3 nM (right).

To investigate the effect of D1 selective agonist or antagonist, we fix [DA]_2_ = 8 nM, [5-HT] = 0.3 nM, [DA] = 3 nM, and vary [DA]_1_ in the range 0–10 nM. We find that smaller [DA]_1_ values (simulating D1 receptor agonist) favors larger firing rate amplitudes and slower oscillations (Figure 10). At intermediate [DA]_1_ values, there is a transition to a smaller oscillation amplitude. Higher [DA]_1_ values eventually shuts off the oscillation behavior via a Hopf bifurcation (at [DA]_1_ = 4.79 nM) with the percentage of active D1 receptors approximately at 1/(1 + exp(1.78667)) = 14.35%. With regard to the neuronal activity, decreasing [DA]_1_ general leads to higher neuronal firing rates. As [DA]_1_ increases, the oscillation amplitude of D1-expressing neurons decreases slower (Figures 10A,C) than that of D2 expressing neurons (Figure 10B), before they all reaches a stable asynchronous tonic state.

**Figure 11:**
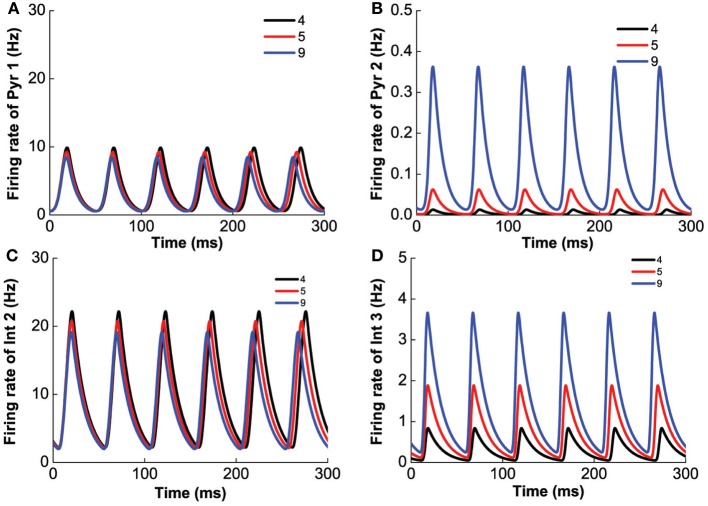
**D2 selective agonist/antagonist affects the oscillation amplitudes of D2-expressing neurons.** [DA]_2_ increases from 4 to 9 nM slightly decrease the frequency of oscillation. **(A)** Firing rate of pyramidal cell expressing D1 and 5-HT1A receptors. **(B)** Firing rate of pyramidal cells expressing D2 and 5-HT1A receptors. **(C)** Firing rate of interneuron expressing D1 and 5-HT2A receptors. **(D)** Firing rate of interneurons expressing D2 and 5-HT1A receptors.

Next, we vary [DA]_2_ while fixing [DA]_1_ = 4 nM, [5-HT] = 0.3 nM, [DA] = 4 nM, to mimic the influence of D2 selective agonist/antagonist on the oscillation. The results are shown in Figure 11. Unlike D1 modulation, no bifurcation happens when we vary [DA]_2_, and the behavior of the network does not change dramatically. The variation of [DA]_2_ only slightly increase the oscillation frequency (Figure 11), but affects the oscillation amplitudes of only D2-expressing neurons (Figures 11B,D).

**Figure 12:**
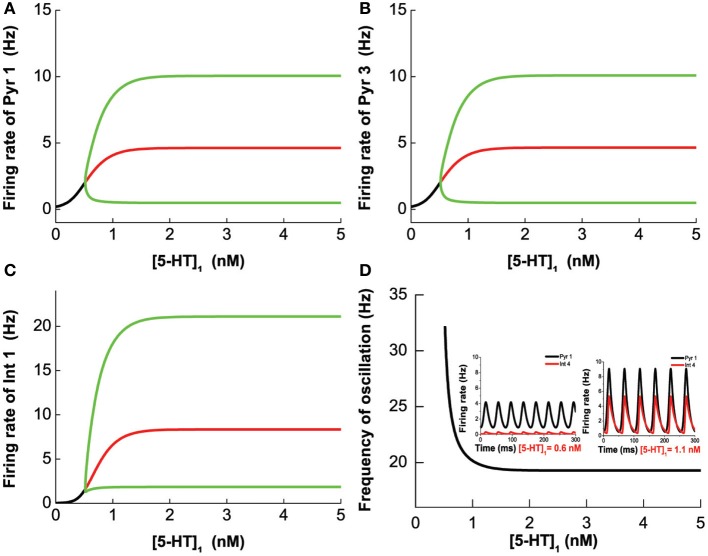
**5-HT1A selective agonist/antagonist effects on the network behavior.** The oscillation merges through Hopf bifurcation at [5-HT]_1_ = 0.516 nM. The amplitude increases with increasing [5-HT]_1_ and reaches a plateau after almost all of 5-HT1A receptor are blocked. Firing rate of pyramidal cell (D1+5-HT1A) **(A)**, pyramidal cell (D2+5-HT1A) **(B)**, and interneuron (D1+5-HT1A) **(C)**. **(D)** Oscillation frequency decreases with increasing [5-HT]_1_ and approaches a stable value. Insets: firing rate timecourse of pyramidal cell (D1+5-HT1A) and interneuron (D2+5-HT2A) with [5-HT]_1_ = 0.6 nM (left) and [5-HT]_1_ = 1.1 nM (right).

To study the effects of selective 5-HT 1A agonist/antagonist, we vary [5-HT]_1_ while fixing [DA]_1_ = 4 nM, [DA]_2_ = 8 nM, [DA] = 4 nM, [5-HT]_2_ = 2 nM, [5-HT] = 0.3 nM. We find that the system becomes oscillatory once [5-HT]_1_ exceeds a critical value (Hopf bifurcation point at [5-HT]_1_ = 0.516 nM). The frequency (amplitude) of the oscillation decreases (increases) with increasing [5-HT]_1_, eventually reaching a robust oscillatory behavior after the 5-HT1A receptors are blocked (Figure 12).

**Figure 13:**
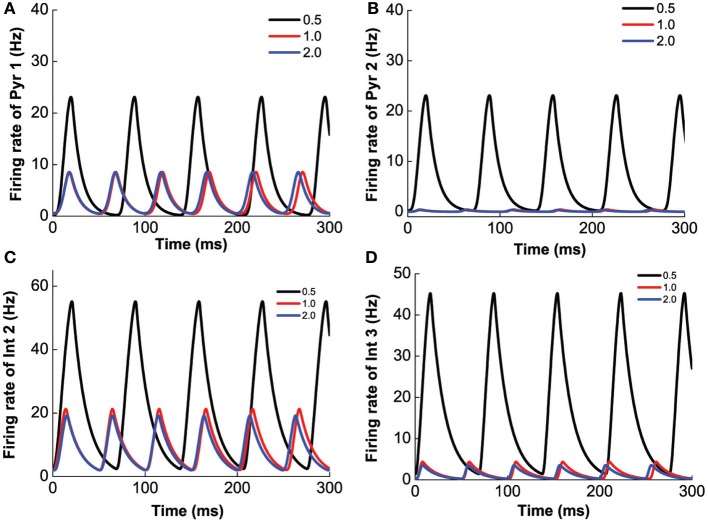
**5-HT2A selective agonist/antagonist on the network behavior.** Increasing [5-HT]_2_ from 0.5 to 2.0 nM, the amplitude of the firing rate oscillation decreases, but the frequency of the oscillation slightly increases. Firing rate of pyramidal cell (D1+5-HT1A) **(A)**, pyramidal cell (D2+5-HT1A) **(B)**, interneuron (D1+5-HT2A) **(C)**, and interneuron (D2+5-HT1A) **(D)**.

Finally, we investigate the modulation of 5-HT2A selective agonist/antagonist by fixing [DA]_1_ = 4 nM, [DA]_2_ = 8 nM, [DA] = 4 nM, [5-HT]_1_ = 1 nM, and varying [5-HT]_2_. Increasing [5-HT]_2_ from 0 to 5 nM, 5-HT2A receptors transit from the activated state to the blocked state. As a result, the amplitude of the oscillation decreases, but the frequency of the oscillation increases (Figure 13). It is to be noted that the different neuronal types are affected differently. For example, the firing rate of pyramidal cells expressing D1 and 5-HT1A receptors decreases to approximately 10 Hz (Figure 13A), but that of pyramidal cells expressing D2 and 5-HT1A (or 5-HT1A and 5-HT2A) receptors decreases to less than 1 Hz (Figure 13B). The firing rate of interneurons expressing D1 and 5-HT1A (or 5-HT2A) decreases to approximately 20 Hz (Figure 13C), and that of interneurons expressing D2 and 5-HT1A (or 5-HT2A) receptors decreases to less than 5 Hz (Figure 13D). As atypical antipsychotic drugs typically block 5-HT2A and D2 receptors (Maher et al., 2002, 2011), we also investigated such drug effects by increasing the values of [5-HT]_2_ and [DA]_2_, but we do not find much difference from that of individually varying [5-HT]_2_ and [DA]_2_.

## 4. Discussion

### 4.1. Summary of results

In this work, we have shown, from single neuron to neuronal circuits, how DA and 5-HT, with their multiple receptors and combinations, can tonically modulate the PFC neural activity, resulting in a variety of complex behaviors.

Due to the different affinities and opposing effects of the 5-HT1A and 2A receptors, the neuronal firing activity of a PFC excitatory neuron coexpressing these two receptors can be inhibited before being enhanced as 5-HT concentration increases. When we extend our analysis to the two-population excitatory-inhibitory neuronal networks, we find that generally, pyramidal cells expressing D1 receptors can provide various interesting network behaviors. In particular, 5-HT and DA can modulate the amplitude and frequency of the network oscillations. Depending on the receptor types expressed by the neurons in the network, 5-HT and DA modulation can cause the oscillations to emerge or cease. Hence, this can result in a finite oscillatory regime, which can create optimal oscillation frequency and amplitude with respect to certain [DA] or [5-HT] level. Moreover, we find that certain combinations of receptors are conducive for the robustness of the oscillatory regime, and for the existence of multiple oscillatory regimes.

The analysis of the two-population model provides us a leverage to understanding the more complex and realistic heterogenous network model. In the heterogeneous network model, the pyramidal cells and interneurons with all the considered combinations of D1, D2, 5-HT1A and 5-HT2A receptors are synaptically coupled. The model reveals that for the network to oscillate, it requires a sufficiently high level of [DA]. At intermediate levels of [DA], an interesting bimodal feature with respect to 5-HT concentration level appears - network oscillation can occur in two separate ranges of 5-HT concentration level. This bimodal feature is largely contributed by the different affinities and opposing effects of 5-HT1A and 5-HT2A receptors, as observed in the two-population model. Very low DA concentration level can suppress the oscillation regardless of the 5-HT level. Finally, we show that selective D1 receptor antagonists (agonists) tend to suppress (enhance) network oscillations, and shift from beta toward gamma band, while selective 5-HT1A antagonists (agonists) act in opposite ways. Selective D2 or 5-HT2A receptor antagonists can lead to decrease in oscillation amplitudes, but only 5-HT2A antagonists can increase the oscillation frequency.

Based on the analysis of the two-population and full network models, a general trend can be observed: the oscillation frequency will decrease if the change causes an overall increase in excitation within the network ([DA]_1↓_, [DA]_2↑_, [5-HT]_1↑_, and [5-HT]_2↓_), and vice versa.

### 4.2. Relations to neuropharmacological drug effects

As mentioned earlier, abnormal beta and gamma band oscillations have been observed in various neurological and neuropsychiatric disorders (Spencer et al., 2003; Cho et al., 2006; Uhlhaas and Singer, 2006, 2010; Basar and Guntekin, 2008; Gonzalez-Burgos and Lewis, 2008; Gonzalez-Burgos et al., 2010). Schizophrenic patients (late responder to antipsychotic drugs) have been shown to have enhanced power in the beta2 (16.5–20 Hz) frequency band in the frontal cortex as compared to controls (Merlo et al., 1998; Venables et al., 2009). Using fluphenazine, an antagonist of both pre- and post-synaptic D2 receptors, beta2 in schizophrenic patients can be reduced (Kleinlogel et al., 1997). In our model, if we only simulate the effect of D2 postsynaptic receptor antagonist, the results is actually an enhancement of beta2 oscillation amplitude (Figure 11). However, antagonist of D2 pre-synaptic receptors can effectively decrease the overall DA concentration level, which can result in a shift in the oscillation frequency out of the beta2 range (Figure 7). Hence the model suggests that the antagonist effects on the D2 presynaptic receptors may be more dominant than the D2 postsynaptic receptors.

In a rat model of Parkinson's disease, beta band oscillation in the frontal cortex is abnormally high compared to controls (Sharott et al., 2005). In that work, the authors showed that administration of apomorphine, a non-selective dopamine agonist which activates both D1- and D2-like receptors (but with higher preference for D2-like receptors), reduces the high beta band power and shifts the oscillation slightly toward higher frequency (from 28 Hz to about 35 Hz). In our model, D2 agonist generally reduces the oscillation amplitude of the beta band (Figure 11), while D1 agonist slightly decreases the oscillation frequency. The former is consistent with the experiment but not the latter. This discrepancy deserves to be further investigated.

Hallucinogens (psychedelics) are agonists of 5-HT2 receptors, enhancing PFC activity and metabolism in humans, and for treatment of psychiatric disorders (Nichols, 2004). It is shown that application of the 5-HT2 agonist (2,5-Dimethoxy-4-iodoamphetamine or DOI) as compared to 5-HT2 antagonist (ketanserin) can lower the power of the beta band in the EEG signal from the frontal cortex of anesthesized rats (Budzinska, 2009). Our model with high [5-HT]_2_ value (mimicking 5-HT2A antagonists) also reduces the oscillation amplitude in the beta band (Figure 13), and thus is consistent with the experiments.

### 4.3. Model limitations and future work

Our modeling approach involves incorporating various experimental data to constrain the model parameters. This includes electrophysiological and pharmacological properties of PFC neurons and synapses, and how these are distinctly modulated by the different DA and 5-HT receptors. Thus, our approach lies more toward biologically constrained firing-rate models (Wong and Wang, 2006; Eckhoff et al., 2011) than abstract connectionist models (Fellous and Linster, 1988). This is a first step toward a systemic understanding of DA and 5-HT comodulation in the PFC. Admittedly, the model has its limitations.

As expected from large-scale biologically based modeling, many model parameters are involved here. We have tried to base as many parameters as possible from experimental data. Some of these parameters are directly obtained or inferred from experimental measurements, while others are based on indirect evidences or assumptions. The extensive investigations on the localization of DA and 5-HT receptors in PFC provided biological plausible proportions of subpopulations of neurons in the PFC network. However, we did not simulate all possible details in the model. For example, we did not include pyramidal cells which coexpress both D1 and D2 receptors. We also did not include PFC neurons which do not express D1, D2, 5-HT1A and 5-HT2A receptors. It remains unknown how these neurons will indirectly affect PFC network behavior upon 5-HT and DA co-modulation. Moreover, DA and 5-HT receptors generally have low and high affinity states, but the present model assume only receptors with high affinity states. In terms of selecting the parameters for the model, we have only chosen a single value for each parameter or variable (e.g., tonic basal [5-HT] in the PFC) within a range of available values identified over various separate experiments. This problem is often encountered when integrating data from multiple sources during model development. Furthermore, oscillations in the cerebral cortex can differ among cortical layers, but the current model does not deal with this issue. The present model also considers only tonic release state, which is more of a resting state and independent of any specific cognitive task.

Despite these limitations and assumptions, it is sometimes advantageous to understand neuromodulation phenomena from simpler to more complex models, teasing apart the contributions of individual components of a system - a key advantage of computational modeling. As we can easily observe, even with such simplified models, the behaviors produced due to the DA-5-HT comodulation are already rather complex. Moreover, the specific [DA]_1_ and [DA]_2_, [5-HT]_1_ and [5-HT]_2_ values only reflect their comparative affinities with DA or 5-HT. Variation of these values while keeping their relative affinities will not dramatically change the network's qualitative behavior under DA and 5-HT co-modulation. That is, their absolute values are not as important as their relative values. A possible extension of our present work would be to explicitly specify the cortical layers, where the latter are known to be distinctively modulated by DA and 5-HT (Wang, 2010). Furthermore, for the model to generate slower oscillations such as theta and other lower frequency bands, and hence directly compare with other experimental data (Benchenane et al., 2010; Puig et al., 2010), the model may require additional slower dynamical features such as GABA_B_-mediated synaptic currents. These concerns will be addressed in future work.

### Conflict of interest statement

The authors declare that the research was conducted in the absence of any commercial or financial relationships that could be construed as a potential conflict of interest.
